# Application of a Simple Parkinson's Disease Risk Score in a Longitudinal Population‐Based Cohort

**DOI:** 10.1002/mds.28127

**Published:** 2020-06-03

**Authors:** Kathrin Marini, Philipp Mahlknecht, Franziska Tutzer, Heike Stockner, Arno Gasperi, Atbin Djamshidian, Peter Willeit, Stefan Kiechl, Johann Willeit, Gregorio Rungger, Alastair J. Noyce, Anette Schrag, Werner Poewe, Klaus Seppi

**Affiliations:** ^1^ Department of Neurology Innsbruck Medical University Innsbruck Austria; ^2^ Department of Neurology Hospital of Bruneck Bruneck Italy; ^3^ Department of Public Health and Primary Care University of Cambridge Cambridge United Kingdom; ^4^ VASCage, Research Centre on Vascular Ageing and Stroke Innsbruck Austria; ^5^ Department of Clinical and Movement Neurosciences University College London Institute of Neurology, University College London London United Kingdom; ^6^ Preventive Neurology Unit Wolfson Institute of Preventive Medicine, Barts and the London School of Medicine and Dentistry, Queen Mary University London United Kingdom

**Keywords:** epidemiology, preclinical, prediagnostic, prodromal Parkinson's disease, risk markers

## Abstract

**Background:**

Identifying individuals at risk of developing Parkinson's disease (PD) is critical to define target populations for future neuroprotective trials.

**Objective:**

The objective of this study was to apply the PREDICT‐PD algorithm of risk indicators for PD in a prospective community‐based study (the Bruneck study), representative of the general elderly population.

**Methods:**

PREDICT‐PD risk scores were calculated based on risk factor assessments obtained at baseline (2005, n = 574 participants). Cases of incident PD were identified at 5‐year and 10‐year follow‐ups. Participants with PD or secondary parkinsonism at baseline were excluded (n = 35). We analyzed the association of log‐transformed risk scores with the presence of well‐established markers as surrogates for PD risk at baseline and with incident PD at follow‐up.

**Results:**

A total of 20 participants with incident PD were identified during follow‐up (11 after 5 years and 9 after 10 years). Baseline PREDICT‐PD risk scores were associated with incident PD with odds ratios of 2.09 (95% confidence interval, 1.35–3.25; *P* = 0.001) after 5 years and of 1.95 (1.36–2.79; *P* < 0.001) after 10 years of follow‐up per doubling of risk scores. In addition, higher PREDICT‐PD scores were significantly correlated with established PD risk markers (olfactory dysfunction, signs of rapid eye movement sleep behavior disorder and motor deficits) and significantly associated with higher probability for prodromal PD according to the Movement Disorder Society research criteria at baseline.

**Conclusions:**

The PREDICT‐PD score was associated with an increased risk for incident PD in our sample and may represent a useful first screening step in future algorithms aiming to identify cases of prodromal PD. © 2020 The Authors. *Movement Disorders* published by Wiley Periodicals, Inc. on behalf of International Parkinson and Movement Disorder Society.

The diagnosis of Parkinson's disease (PD) is preceded by a prodromal phase^1^, ^2^ where neuropathology and neuronal dysfunction have started in multiple sites of the central and peripheral autonomic nervous system.^3^, ^4^ Identifying patients in these earliest stages of the disease is a key priority in current PD research and critical for the implementation of future disease‐prevention trials.^5^

A number of risk factors and prodromal symptoms of PD have been identified,^5^ and a few algorithms to quantify PD risk have been proposed, but their performance in the general elderly population needs further study.^2^

The PREDICT‐PD study algorithm is based on a comprehensive meta‐analysis of early nonmotor features and risk factors.^6^ It was initially implemented in an online cohort, showing an association with olfactory performance, motor deficits, and signs of probable rapid eye movement sleep behavior disorder (RBD) as disease‐state surrogates in a cross‐sectional analysis and with incident PD during follow‐up over 3 years with a hazard ratio (HR) of 4.39 (95% confidence interval [CI], 1.03–18.68).^7^ A validation attempt in the population‐based Rotterdam study showed an association of the risk score with incident PD with a HR of 1.30 that did not significantly improve classification and discrimination beyond age and sex.^8^

The simple, questionnaire‐based approach of the PREDICT‐PD algorithm would make an attractive tool for future population‐based risk screening, Therefore, we assessed the association of the PREDICT‐PD algorithm with incident PD in the longitudinal Bruneck study cohort, a sample of the general elderly population, during an overall follow‐up period of 10 years.

## Methods

### Study Design and Population

Assessments were carried out in the Bruneck study, a prospective population‐based study on cardiovascular and neurological diseases initiated in 1990.^9^, ^10^ Their follow‐up assessment of 2005 included early nonmotor features and risk markers^10^ required for the PREDICT‐PD algorithm and was used as a baseline for this analysis. The study was approved by the local institutional review board. All participants gave written informed consent, and the assessments were carried out in accordance with the Declaration of Helsinki.

### Baseline and Follow‐up Assessments

In 2005, 574 participants (aged 55–94 years) underwent a standardized interview and neurological examination including the Unified PD Rating Scale motor section (UPDRS Part III). Medical history, medication, and family history with a focus on movement disorders and possible causes for secondary parkinsonism were recorded. Assessments were performed by 2 neurologists with special expertise in the field of movement disorders.

Follow‐up examinations took place in 2010 after a median of 5.0 years (range, 4.9–5.0) and in 2016 after a median of 10.4 years (range, 10.4–10.5). Assessments followed a similar protocol as in 2005 and were performed by 2 neurologists blinded to baseline risk markers. Incident PD cases were identified as per UK PD Society Brain Bank criteria.^11^ Data from 465 (86.3%) participants in 2010 and 341 (63.3%) in 2016 were available for analysis.

For a detailed flowchart of the study population including deceased participants and differences of participants according to follow‐up status, see Supplementary Figure 1 and Supplementary Table 1.

### Ascertainment of Early Nonmotor Features and PD Risk Markers

Following factors for the calculation of the PREDICT‐PD algorithm were available: age, sex, smoking status, coffee use, hypertension, use of nonsteroidal anti‐inflammatory drugs, calcium channel blockers, beta blockers, alcohol consumption, constipation, depression and/or anxiety, known history of head injury, and a first‐degree relative with history of PD (Table 1). Application of the algorithm was performed analogous to the description in the original publication of the PREDICT‐PD score.^12^

**TABLE 1.** Participant's characteristics at baseline

Factor	All Participants, n = 539	Incident PD, n = 20	Predefined RR/OR from Systematic Review^6^
Age^a^	69.1 ± 9.4 67.2 (61.4–76.2)	70.2 ± 7.170.5 (63.4–76.2)	See Methods section
Female^b^	290 (53.7%)	9 (45.0%)	0.67
Smoker^b^			
Current	73 (13.5%)	2 (10.0%)	0.44
Former	162 (30.0%)	8 (40.0%)	0.78
Never	305 (56.5%)	10 (50.0%)	1.00
Coffee use^b^	466 (86.3%)	18 (90.0%)	0.67
Hypertension^b^	385 (71.3%)	14 (70.0%)	0.74
NSAID use^b^	56 (10.4%)	0 (0.0%)	0.83
CCB use^b^	72 (13.3%)	3 (15.0%)	0.90
Alcohol^b^	351 (65.0%)	14 (70.0%)	0.90
Beta blocker use^b^	74 (13.7%)	1 (5.0%)	1.28
Head injury^b^	40 (7.4%)	1 (5.0%)	1.58
Depression/anxiety^b^	88 (16.3%)	7 (35.0%)	1.86
Constipation^b^	86 (15.9%)	5 (25.0%)	2.34
Family history of PD^b^	41 (7.6%)	5 (25.0)	4.45

a Quantitative results are reported in mean with standard deviation and medians (25th–75th percentile).

b Binominal variables are given in number and percentage of the respective category.

PD, Parkinson's disease; RR, risk ratio; OR, odds ratio; NSAID, nonsteroidal anti‐inflammatory drugs; CCB, calcium channel blocker.

The following established risk markers (“intermediate” surrogate outcomes in the original PREDICT‐PD study)^12^ were available: olfactory loss, assessed with the 12‐item sniffin' sticks identification test; probable RBD, assessed throughout the RBD Screening Questionnaire; possible subthreshold parkinsonism using the motor section of the UPDRS (Part III); and substantia nigra (SN) echogenicity on transcranial sonography. The distribution of these risk markers and of the Movement Disorder Society (MDS) research criteria for prodromal PD^13^ at the same time point in the Bruneck cohort and their performance in predicting incident cases of PD is published elsewhere.^10^, ^14^

A detailed description of the ascertainment of all markers and respective cut‐offs is available in the Supplementary Methods.

### Statistical Analysis

As nonnormal distributions for continuous quantitative variables were shown throughout, group comparisons were performed with the Mann‐Whitney *U* test. Significance levels for binary data were assessed with the chi‐square test. Cross‐sectional correlation analysis between log_2_‐transformed risk scores (expressed as odds) and established PD risk markers at baseline was performed using the Spearman rank test. The predictive value of the risk scores for cases of incident PD at follow‐up was examined using binary logistic regression analysis for the PREDICT‐PD log odds, and the results are given in odds ratios (ORs) and 95% CIs. Log_2_‐transformation was used following the example of the original PREDICT‐PD cohort in order to render results comparable and allow better interpretation of risk estimates. Statistical analysis was performed using SPSS version 24 for Windows (IBM Corp., Armonk, NY). The significance level was set at a 2‐sided *P* value of ≤0.05.

## Results

Of the 574 baseline participants, 35 had idiopathic PD or secondary parkinsonism and were excluded from the analysis. Table 1 depicts characteristics of the remaining 539 included participants. In the cross‐sectional correlation analysis between established PD risk markers and risk scores we found a weak but significant correlation of PREDICT‐PD risk scores with the sniffin' sticks identification test scores (*r*_s_ = −0.186, *P* < 0.001), RBD Screening Questionnaire scores (*r*_s_ = 0.180, *P* < 0.001), and UPDRS Part III scores (*r*_s_ = 0.176, *P* < 0.001) (Supplementary Fig. 2), but not with SN echogenicity. In addition, the PREDICT‐PD risk scores were significantly higher in the participants with a higher probability of prodromal PD as per MDS research criteria (e.g. 1:33 [1:89–1:17] for those with probabilities above the cut off of ≥80% versus 1:110 [1:207–1:63] for those with <80% probability for prodromal PD, *P* = 0.001; Supplementary Table 2).

A total of 20 participants were identified with incident PD at follow‐up, 11 after 5 years and 9 after 10 years. Baseline PREDICT‐PD risk scores were significantly higher in the participants with PD after 5 years and for the whole 10‐year period of follow‐up (Table 2). Baseline PREDICT‐PD risk scores were associated with incident PD with ORs of 2.09 (95% CI, 1.35–3.25; *P* = 0.001) after 5 years and of 1.95 (95% CI, 1.36–2.79; *P* < 0.001) after 10 years of follow‐up per the doubling of risk scores. The results remained significant even when using risk scores without age and sex (Supplementary Table 3).

**TABLE 2.** Distribution of baseline risk scores and association with incident PD at follow‐ups

Follow‐up	Incident PD/PD Free, n	PREDICT‐PD Risk Scores of PD‐Free Participants^a^	PREDICT‐PD Risk Scores of Incident PD Cases^a^	*P* Value^b^	Log Risk Given in OR (95% CI)^c^ *P* Value
Baseline	0/539	1:109 (1:204–1:62)	–	–	–
0–5 y	11/451	1:116 (1:222–1:70)	1:44 (1:131–1:19)	0.007	2.09 (1.35–3.25); 0.001
5–10 y	9/321	1:141 (1:270–1:81)	1:76 (1:161–1:53)	0.094	1.50 (0.88–2.54) 0.134
0–10 y	20/321	1:141 (1:270–1:81)	1:71 (1:128–1:31)	0.001	1.95 (1.36–2.79) < 0.001

a Distribution of PREDICT‐PD odds is given in median with 25th and 75th confidence intervals.

b Significance levels for distributions of risk scores were calculated using the Mann‐Whitney *U* test as data were not normally distributed.^c^Binary logistic regression analysis of log odds was used to calculate ORs and 95% CIs. ORs are given for a 1‐unit change in log risk scores.

PD, Parkinson's disease; OR, odds ratio; CI, confidence interval.

## Discussion

In this study, we applied the PREDICT‐PD algorithm, a simple questionnaire‐based screening tool aiming to identify participants with increased PD risk in a longitudinal sample of the general elderly population. The PREDICT‐PD study first employed the algorithm in an online cohort with follow‐ups for future PD diagnosis over 3 years, where it predicted incident PD with a HR of 4.39 and correlated with established markers of PD.^7^ An application of this score performed in a longitudinal sample of the population‐based Rotterdam study showed an association of the risk score with incident PD with a HR of 1.30 that did not significantly improve classification and discrimination beyond age and sex.^8^ However, several factors were missing from their calculations or assessed with surrogate markers (eg, constipation). As the scales used for HR calculation in these 2 applications differed (log odds of risk scores vs. transformation of risk scores in *z* scores), a direct comparison of HRs is difficult.

In our sample, we found a significant association of higher risk scores at baseline with a future diagnosis of PD with an OR of 2.09 after 5 and 1.95 after 10 years of follow‐up. These results survived even after omitting age and sex from the score, which is encouraging in light of the ambiguous results in the Rotterdam study. In addition, risk scores significantly correlated with established PD risk markers (olfactory dysfunction, signs of probable RBD, and subtle motor impairment) and were associated with higher posttest probability for prodromal PD as per MDS research criteria at baseline. It should be noted that our data do not allow calculation of HR, as the exact time of conversion was unknown because of the 5‐year follow‐up intervals. However, OR and HR can be comparable at a low incidence rate,^15^, ^16^ which is the case in our cohort and the original PREDICT‐PD cohort. The difference in magnitude of risk estimates in the original PREDICT‐PD online cohort compared with the Bruneck cohort (HR 4.39 vs. OR 2.09) might be explainable because of the rather wide but overlapping CIs related to the small number of PD cases in both samples, different follow‐up times, and differences in recruitments (online cohort in PREDICT‐PD vs. unselected elderly sample).

Distribution of the various risk markers and early nonmotor features as well as age and sex were mostly comparable with the original cohort studied by Noyce and colleagues.^12^ Differences were observed for positive family history for PD (8% vs. 20%), which might reflect differences in the recruitment strategy between both studies. A positive family history of PD has been found in approximately 4% of unselected population samples,^17^ similar to the Bruneck study. Baseline hypertension was much more common in this population compared with the PREDICT‐PD study (76% vs. 25%). The Bruneck study used a precise definition of hypertension based on self‐reported diagnosis and concomitant medication against hypertension as well as 3 separate blood pressure recordings at baseline (see Supplementary Methods), and its prevalence is in line with the results of other population‐based cohorts.^18^

An interesting detail concerns the prediction differences for converters identified after the first as compared with the second 5‐year interval. The OR for this second group was slightly lower at 1.50 and not statistically significant. This observation could point to a declining predictive accuracy of the model over time and is in line with the fact that different prodromal nonmotor and motor manifestations occur at different time intervals prior to the onset of motor symptoms.^2^ As the time of onset of defining motor features of PD approaches, more prodromal markers will have developed such that the identification of true cases of prodromal PD will become more accurate closer to the clinical onset of disease.

The PREDICT‐PD score was developed as an online tool only including items that can be assessed remotely and therefore does not require in‐person examinations. It did not include the established and stronger “intermediate” markers shown to clearly associate to a substantially increased PD risk^2^, ^14^ that could theoretically also be remotely assessed. Instead, these markers were used in the original publication as surrogate markers until sufficient incident cases had occurred during the follow‐up of 3 years, but the group very recently reported that the inclusion of these markers into an updated algorithm may boost its performance.^19^ The PREDICT‐PD approach models risk as a continuum of odds rather than a probability and in that way differs from the Movement Disorder Society prodromal PD score. The latter also includes other factors that can only be assessed in person including clinical examination by trained health professionals (eg, the UPDRS), sonography, or DAT‐SPECT.^20^ Its predictive value has been evaluated in several different cohorts.^10^, ^21^, ^22^ In contrast, the PREDICT‐PD score may serve as a simple first screening step that could also be used online, filtering participants suitable for further in‐person assessments. Participants with an elevated risk may then consecutively be invited for further assessment steps of increasing specificity, aiming to identify those who will finally go on to develop PD. Such assessments may include genetic testing, polysomnography, transcranial sonography, or dopamine transporter imaging, which are more costly and time‐consuming. They may therefore only be used in a more defined group of participants, as envisaged for future risk screening programs such as in the Parkinson's Progression Markers Initiative (PPMI) 2.0 study.^23^

Our study has limitations. The small incidence of case numbers leads to wide confidence intervals and may also contribute to differences observed in risk estimates of the first versus the second 5‐year follow‐up interval. Despite the fact that established criteria for a PD diagnosis were applied by movement disorders specialists, misdiagnosis cannot be excluded. The long follow‐up of 10 years in an unselected population‐based sample represents a strength of our study as does the in‐person assessments by movement disorder specialists blinded to the baseline results, adding to the validity of this analysis. Although our results support the usefulness of the PREDICT‐PD algorithm as a possible screening tool for prodromal PD, large (online) studies will be needed to finally determine its value.

## Author Roles

(1) Research Project: A. Conception, B. Designing the Bruneck Study, C. Obtaining Funding, D. Organization, E. Acquisition of Data; (2) Statistical Analysis: A. Design, B. Execution, C. Interpretation; (3) Manuscript: A. Drafting of the Manuscript, B. Revisions.

K.M.: 2A, 2B, 2C, 3A, 3B

P.M.: 1A, 1B, 1C, 1D, 1E, 2A, 2C, 3A, 3B

F.T.: 2C, 3B

H.S.: 1E, 3B

A.G.: 1B, 1E, 3B

A.D.: 1E, 3B

P.W.: 2C, 3B

S.K.: 1B, 1C, 1D, 1E, 2C, 3B

J.W.: 1B, 1C, 1D, 1E, 3B

G.R.: 1E, 3B

A.J.N.: 1A, 2C, 3B

A.S.: 1A, 2C, 3B

W.P.: 1A, 1C, 2C, 3B

K.S.: 1A, 1B, 1E, 2A, 2C, 3B

## Financial Disclosures of all authors (for the preceding 12 months)

A.J.N. reports a salary from Barts Health NHS Trust and from the Barts Charity to support the Preventive Neurology Unit at the Wolfson Institute of Preventive Medicine, QMUL; grants from Parkinson's UK, the Leonard Wolfson Experimental Neurology Centre, the UCL Movement Disorders Centre, and the Virginia Kieley Benefaction; and honoraria/consultancy fees from Britannia Pharmaceuticals, Global Kinetics Corporation, Profile Pharmaceuticals, Bial Pharmaceuticals, Biogen, and F. Hoffmann‐La Roche. A.S. reports research funding or support from University College London, National Institute of Health, National Institute for Health Research ULCH Biomedical Research Centre, the International Parkinson and Movement Disorder Society, the European Commission, Parkinson's UK, Royal Free Hospital charity, GE Healthcare, and the Economic and Social Research Council; honoraria for consultancy from Biogen, Roche, Bial, GE Healthcare; license fee payments from the University College London for the MSA‐QoL, PSP‐QoL, and PQolCarers; and royalties from Oxford University Press. K.S. reports grants from Oesterreichische Nationalbank, FWF Austrian Science Fund, Michael J, Fox Foundation, and International Parkinson and Movement Disorders Society and consultancy and lecture fees from Boehringer‐Ingelheim, UCB, Lundbeck, AOP Orphan Pharmaceuticals AG, Roche, and Teva. W.P. reports consultancy and lecture fees in relation to clinical drug programs from AbbVie, AstraZeneca, BIAL, Biogen, Cynapsus, Britannia, Grünenthal, Intec, Ipsen, Lundbeck, Merz Pharmaceuticals, Novartis, Neuroderm, Orion Pharma, Prexton, Teva, UCB, and Zambon and royalties from Thieme, Wiley Blackwell, Oxford University Press, and Cambridge University Press. All other authors have nothing to report.

## Supporting information


**Appendix** **S1**: Supporting Information
