# Supplementary material for: Application of a Simple Parkinson's Disease Risk Score in a Longitudinal Population‐Based Cohort
Source: Mov Disord. 2020 Jun 3;35(9):1658–62. doi: 10.1002/mds.28127 (PMC7540037; doi:10.1002/mds.28127)
Supplement: Supplementary file 1 — Appendix S1: Supporting Information [file MDS-35-1658-s001.docx]

**SUPPLEMENTARY MATERIALS**

**APPLICATION OF A SIMPLE PARKINSON’S DISEASE RISK SCORE IN A LONGITUDINAL POPULATION BASED COHORT**

**SUPPLEMENTARY METHODS**

**1. Study Design**

This investigation was part of the Bruneck Study, a prospective population-based study to assess the epidemiology of cardiovascular and neurological disease. ^1,2^ Bruneck is a small town located in the alpine region of northern Italy. The population of Bruneck is exclusively white and of heterogeneous geographic origin with sizeable segments of Austro-German or Italian background. Population mobility within the survey area is 0.2% per year. The study population was recruited in 1990 as an age- and sex-stratified random sample of all inhabitants of Bruneck aged 40–79 years. A total of 1,000 persons were selected for inclusion from the official population register by a computer-based random number generator. The participants were split into age groups formed by decades (125 women and 125 men of every decade from the ages of 40–49 to 70–79 years). 919 of 1000 selected subjects agreed to participate in this prospective study. Follow-up assessments were carried out every 5 years. Assessments of a variety of clinical signs and risk markers relevant for movement disorders and Parkinson’s disease (PD) have been added to the protocol from the 2000 assessment onwards ^2^ and the Bruneck Study cohort from the 2005 assessment was also part of the PRIPS study prospectively establishing Substantia Nigra (SN) hyperechogenicity on Transcranial Sonography (TCS) as a risk marker for Parkinson’s Disease (PD). ^3,4^

**2. Ascertainment of risk indicators included in the PREDICT-PD algorithm**

Data for the calculation of the PREDICT-PD score were obtained during the Bruneck Study follow-up visit in 2005, which forms the baseline for the present analysis. In addition to the neurological part of the Bruneck Study, which has a focus on movement disorders, data for the algorithm were also retrieved from the “general part” of the study in order to be as comprehensive as possible as outlined below. As the study was performed in the hospital of Bruneck, additional data was available through the electronic medical records. Calculation was performed following the equation in the original manuscript of the PREDICT-PD study. ^5^

All markers except for erectile dysfunction were available. Markers were classified in binary terms (yes/no for presence or absence of the specific risk marker). Where presence of risk factors could not be unambiguously determined and in cases of missing data, variables were attributed with the factor 1.

Age as a risk factor for the development of PD was considered using the equation for age-related PD risk described in the original PREDICT-PD publication, which is based on results of the Physician’s health study. ^6^ Gender as a risk factor was considered as described in the original algorithm. ^7^

Factors, which lowered the result in the PREDICT-PD algorithm (therefore decreased the risk for future PD) were assessed as following: Smoking status was subdivided in non-smoker, current or former smoker. Caffeine use was defined as consumption of ≥ 1 cup of coffee per day. Alcohol consumption was assessed in gram per day and confirmed for alcohol use > 1 gram per day. Hypertension was measured threefold at the baseline assessment and defined as a mean systolic blood pressure > 140 mmHg or a mean diastolic blood pressure > 90mmHg. Furthermore, self-reported hypertension and use of antihypertensive drugs in the subject’s concomitant medications were defined as positive criteria for the application of the respective risk factor.

NSAID, calcium channel blocker and beta blocker use could be extracted from the general part of the Bruneck Study, where concomitant medication was assessed. NSAID included non-selective as well as COX-2-selective agents (e.g. ibuprofen, paracetamol, acetylsalicylic acid, diclofenac, metamizole, etoricoxib, celecoxib).

Factors increasing the PD risk were assessed in the following way: Family history of PD was self-reported by participants. As 2005 assessment was not asking for family history of PD, we used information gathered during the 2008 interim evaluation of the cohort, as this risk marker can be considered mostly time-independent disease. Constipation criteria were applied according to the frequency of spontaneous bowel movement. A frequency of < 1 per day was denoted as low bowel movement and therefore an indicator for constipation. Furthermore, use of laxatives was taken as a surrogate marker for constipation. Diagnosis of past head injury was available through the subject’s electronic medical records of the Bruneck hospital. A positive history of head injury was applied for a diagnosis of fractures of the head, commotio cerebri or traumatic brain injury. Finally, anxiety and depression were assessed applying item 26 (“How often do you have negative feelings such as blue mood, despair, anxiety or depression?”) of the multidimensional World Health Organization Quality of Life BREF (WHOQoL-BREF) instrument. An answer of ≥4 (“very often” or “always”) was considered as a positive cut-off. Furthermore, antidepressant drugs use in the medical history was considered as surrogative for depression.

Erectile dysfunction was not assessed in the 2005 follow-up. Severe erectile dysfunction requiring medical intervention was only indirectly assessable via the intake of phosphodiesterase type 5 inhibitors and in the 2005 Bruneck Study population no subject was using the respective medication (or didn’t provide information for the medical record).

**3. Ascertainment of established risk markers for cross-sectional analysis**

We performed cross-sectional analysis between the subjects’ calculated risk score and established risk markers for PD at baseline as a preliminary outcome measure. Following markers were used: olfactory performance, probable REM-sleep behaviour disorder (RBD), worse results in the motor examination of the Unified Parkinson’s Disease Rating Scale (UPDRS Part III) as a surrogate for possible subthreshold parkinsonism, ^8^ and finally substantia nigra echogenicity on transcranial sonography (TCS).

Olfactory performance was assessed using the 12-item Sniffin Sticks odor identification test (SS-12; Burghart Medizintechnik, Germany). ^9^ For the ascertainment of probable RBD, subjects answered the 10 question RBD Screening Questionnaire (RBDSQ). ^10^ For the evaluation of participant’s motor performance, part III of the UPDRS was assessed and the total sum score calculated. Substantia nigra hyperechogenicity was examined by a neurologist with relevant experience in TCS (HS), who was blinded to the results of the clinical examination. A 2.5 MHz transducer (Logiq 7; General Electrics, Milwaukee, WI) was used for sonography as described elsewhere. 85 participants had insufficient bone window to perform TCS. ^11^ Intra- and interrater reliability of TCS results in this cohort were excellent as previously published (all intraclass correlation coefficients ≥0.91, p<0.001). ^11,12^

**Supplementary Table 1:** Distribution of demographic data in subjects attending follow-ups visits versus respective subjects lost to follow-up.

|  | **Baseline** | **Follow-up 0-10 years** | | | |
| --- | --- | --- | --- | --- | --- |
|  | **(n=539)** | **FU attended (n=341)** | **Lost to FU (n=198)** | **p-values** |  |
| Age^a^ | 67.2 (61.4–76.2) | 64.6 (59.7 – 70.6) | 75.6 (68.8 – 82.0) | < 0.001 |  |
| Sex (m/f)^b^ | 250 (46.3%) / 290 (53.7 %) | 173 (50.7 %) / 168 (49.3 %) | 77 (38.7 %) / 122 (61.3 %) | 0.007 |  |
| UPDRS III score^a^ | 0.0 (0.0 – 0.0) | 0.0 (0.0 - 0.0) | 0.0 (0.0 – 0.1) | 0.002 |  |
| PREDICT-PD score^a^ | 1:109 (1:204 – 1:62) | 1:138 (1:262 – 1:77) | 1:83 (1:127 – 1:50) | < 0.001 |  |

Abbreviations: FU = Follow-Up; PD = Parkinson’s disease; UPDRS = Unified Parkinson's Disease Rating Scale.

^a^ Quantitative results are reported in mean with standard deviation and medians (25^th^-75^th^ percentile). Significance levels were calculated using x^2^-test.

^b^ Binominal variables are given in number (n) and percent of the respective category. As data was not-normally distributed, significance levels were calculated with the Mann-Whitney U test (2-sided).

**Supplementary Table 2:** Associations between baseline PREDICT-PD risk scores and probability of prodromal PD (posttest probability ≥ 80%, ≥ 50% and ≥ 30%).

| **posttest probability** | **affected subjects**  **(n, (%))** | **PREDICT-PD risk scores^a^** | | |
| --- | --- | --- | --- | --- |
|  |  | Posttest probability  above cut-off | Posttest probability  below cut-off | **p-value** |
| ≥ 80% | 12 (2.2%) | 1:33 (1:89 – 1:17) | 1:110 (1:207 – 1:63) | **0.001** |
| ≥ 50% | 30 (5.6%) | 1:43 (1:114 – 1:21) | 1:110 (1:209 – 1:65) | **< 0.001** |
| ≥ 30% | 55 (10.2%) | 1:45 (1:91 – 1:25) | 1:114 (1:219 – 1:68) | **< 0.001** |

Abbreviations: n = number, PD = Parkinson’s disease.

^a^ PREDICT-PD risk scores are given in median with 25^th^ and 75^th^ percentile. As data was not-normally distributed, significance levels were calculated with the Mann-Whitney U test (2-sided)

**Supplementary Table 3:** Contribution of age and sex on performance of the algorithm: logistic regression analysis of a PREDICT-PD model omitting age and sex.

| **Follow-up** | **Incident PD / PD-free (n)** | **PREDICT-PD log odds excluding age and sex** | |
| --- | --- | --- | --- |
|  |  | OR (95% CI) ^a^ | p-value |
| 0-5 years | 11 / 451 | **1.98** (1.17 – 3.35) | **0.010** |
| 5-10 years | 9 / 321 | 1.58 (0.81 – 3.07) | 0.179 |
| 0-10 years | 20 / 321 | **1.85** (1.20 – 2.87) | **0.006** |

Abbreviations: CI = confidence interval; OR = odds ratio; PD = Parkinson’s Disease.

^a^ Binary logistic regression analysis of log_2_-transformed risk scores was used to calculate OR and 95% CI. OR are given for a 1-unit change in log risk scores.

**Supplementary Figure 1:** Flowchart of the Bruneck Study: participant numbers, dropout rates and diagnosis of incident PD for the respective analysis


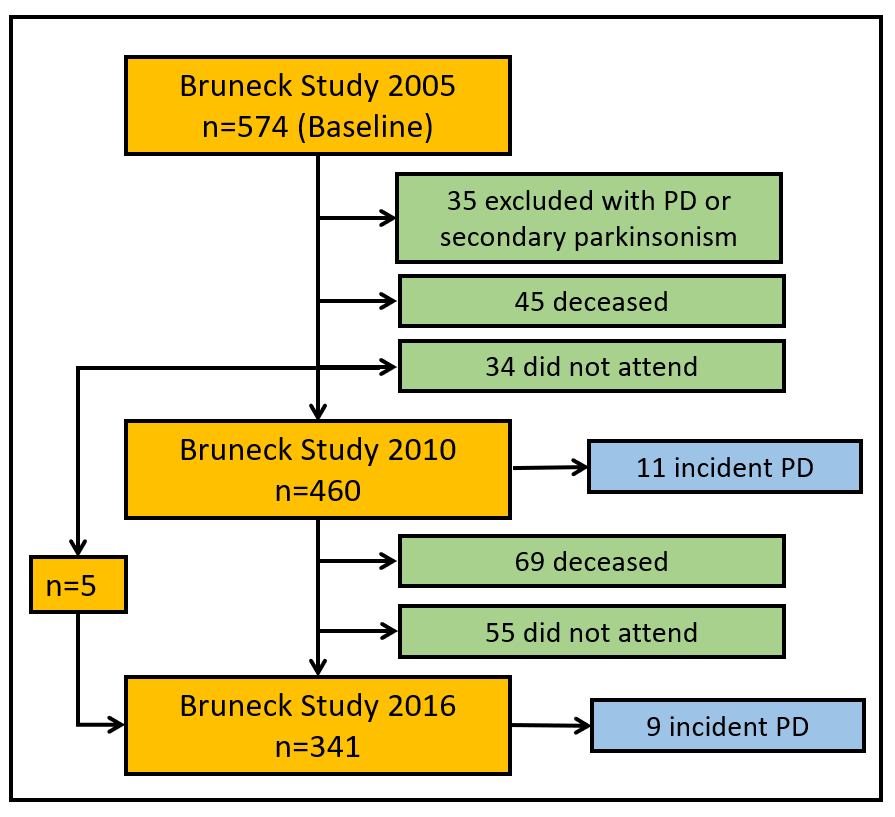


**Supplementary Figure 2:** Established PD risk markers at baseline plotted against estimated risk of PD.


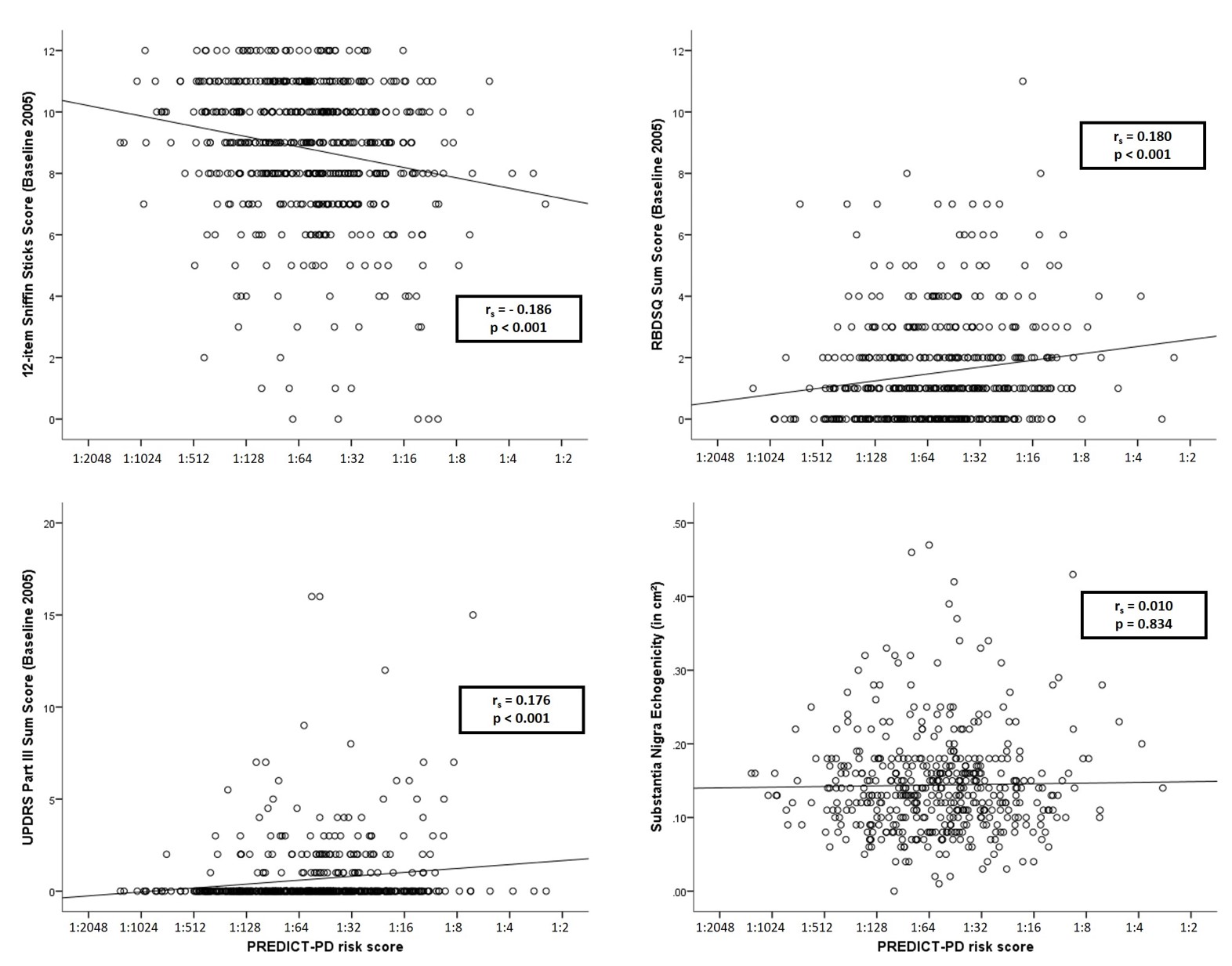


Abbreviations: PD = Parkinson’s Disease; UPDRS = Unified Parkinson’s Disease Rating Scale; RBDSQ = REM sleep behaviour disorder screening questionnaire; r_s_ = Spearman’s coefficient.

**References**

1. Kiechl S, Willeit J. In a Nutshell: Findings from the Bruneck Study. Gerontology. 2019;65(1):9–19.

2. Wenning GK, Kiechl S, Seppi K, et al. Prevalence of movement disorders in men and women aged 50-89 years (Bruneck Study cohort): A population-based study. Lancet Neurol. 2005;4(12):815–820.

3. Berg D, Behnke S, Seppi K, et al. Enlarged hyperechogenic substantia nigra as a risk marker for Parkinson’s disease. Mov Disord. 2013;28(2):216–9.

4. Berg D, Godau J, Seppi K, et al. The PRIPS study: Screening battery for subjects at risk for Parkinson’s disease. Eur J Neurol. 2013;20(1):102–8.

5. Noyce AJ, Bestwick JP, Silveira-Moriyama L, et al. PREDICT-PD: Identifying risk of Parkinson’s disease in the community: methods and baseline results. J Neurol Neurosurg Psychiatry. 2014;85(1):31–7. .

6. Driver JA, Logroscino G, Gaziano JM, Kurth T. Incidence and remaining lifetime risk of Parkinson disease in advanced age. Neurology. 2009;72(5):432-8.

7. Wooten GF, Currie LJ, Bovbjerg VE, Lee JK, Patrie J. Are men at greater risk for Parkinson’s disease than women? J Neurol Neurosurg Psychiatry. 2004;75(4):637–9.

8. Berg D, Postuma RB, Adler CH, et al.. MDS Research Criteria for Prodromal Parkinson’s Disease. 2015;30(12):1600-11.

9. Hummel T, Kobal G, Gudziol H, Mackay-Sim A. Normative data for the “Sniffin’’ Sticks" including tests of odor identification, odor discrimination, and olfactory thresholds: An upgrade based on a group of more than 3,000 subjects. Eur Arch Oto-Rhino-Laryngology. 2007;264(3):237–43.

10. Stiasny-Kolster K, Mayer G, Schäfer S, Möller JC, Heinzel-Gutenbrunner M, Oertel WH. The REM sleep behavior disorder screening questionnaire - A new diagnostic instrument. Mov Disord. 2007;22(16):2386–93.

11. Mahlknecht P, Seppi K, Stockner H, et al. Substantia nigra hyperechogenicity as a marker for Parkinson’s disease: A population-based study. Neurodegener Dis. 2013;12(4):212–8.

12. Mahlknecht P, Stockner H, Nocker M, et al. A follow-up study of substantia nigra echogenicity in healthy adults. Mov Disord. 2012;27(9):1196–7.
